# Shotgun-metagenomics based prediction of antibiotic resistance and virulence determinants in *Staphylococcus aureus* from periprosthetic tissue on blood culture bottles

**DOI:** 10.1038/s41598-021-00383-7

**Published:** 2021-10-21

**Authors:** Adriana Maria Sanabria, Jessin Janice, Erik Hjerde, Gunnar Skov Simonsen, Anne-Merethe Hanssen

**Affiliations:** 1 Research Group for Host-Microbe Interaction, Department of Medical Biology, Faculty of Health Sciences, UiT – The Arctic University of Norway, Tromsø, Norway; 2 Norwegian Advisory Unit on Detection of Antimicrobial Resistance, Department of Microbiology and Infection Control, University Hospital of North Norway, Tromsø, Norway; 3 Centre for Bioinformatics, Department of Chemistry, UiT – The Arctic University of Norway, Tromsø, Norway; 4 Department of Microbiology and Infection Control, University Hospital of North Norway, Tromsø, Norway

**Keywords:** Computational biology and bioinformatics, Microbiology, Diseases

## Abstract

Shotgun-metagenomics may give valuable clinical information beyond the detection of potential pathogen(s). Identification of antimicrobial resistance (AMR), virulence genes and typing directly from clinical samples has been limited due to challenges arising from incomplete genome coverage. We assessed the performance of shotgun-metagenomics on positive blood culture bottles (n = 19) with periprosthetic tissue for typing and prediction of AMR and virulence profiles in *Staphylococcus aureus*. We used different approaches to determine if sequence data from reads provides more information than from assembled contigs. Only 0.18% of total reads was derived from human DNA. Shotgun-metagenomics results and conventional method results were consistent in detecting *S. aureus* in all samples. AMR and known periprosthetic joint infection virulence genes were predicted from *S. aureus*. Mean coverage depth, when predicting AMR genes was 209 ×. Resistance phenotypes could be explained by genes predicted in the sample in most of the cases. The choice of bioinformatic data analysis approach clearly influenced the results, i.e. read-based analysis was more accurate for pathogen identification, while contigs seemed better for AMR profiling. Our study demonstrates high genome coverage and potential for typing and prediction of AMR and virulence profiles in *S. aureus* from shotgun-metagenomics data.

## Introduction

*Staphylococcus aureus* is an important opportunistic pathogen considered as the most common cause of periprosthetic infections (PIs)^1–5^. The emergence and spread of resistance pose an increasing threat to public health, in particular, methicillin-resistant *S. aureus* (MRSA)^6^. The success of *S. aureus* as a pathogen is in part due to its ability to develop resistance to a wide variety of antimicrobial compounds. Additionally, *S. aureus* can adapt to a biofilm mode of growth whereby infections become persistent and recurrent, particularly in association with prosthetic implants^7^.

Microbiological diagnosis of PJI is challenging. A variety of tools are available for facilitating the diagnosis of PJI, including emerging technologies such as metagenomic approaches^8^. The use of shotgun-metagenomics (SMg) for the analysis of clinical specimens has emerged as a promising approach for pathogen identification, antimicrobial resistance (AMR) identification and outbreak investigation in clinical microbiology laboratories. This approach has been used for the analysis of different types of clinical specimens, including samples related to PJI, e.g. synovial fluid^9,10^, sonication fluid^11–14^ and tissue^15^, mainly for the identification of pathogens.

The advantage of using direct material in SMg is mainly short turnaround time for identification of pathogens, and the use of e.g. Nanopore sequencing is very promising for rapid identification of pathogens within 4–24 h^14,16,17^. However, in these studies, extracted DNA is contaminated with a high concentration of human DNA while the bacterial DNA yield is very low, and thus, leading to a low number of bacterial reads. In a previous study, we showed that SMg performed directly on positive blood culture bottles (BCBs) inoculated with periprosthetic tissue (PT), is a convenient method to identify potential pathogens causing PJI^18^. However, beyond the identification of pathogens, SMg provides unlimited access to other clinically relevant genomic features such as antibiotic resistance, virulence genes profiles and strain-level typing^19,20^.

Currently, SMg is considered in its infancy for pathogen characterization, including inference of antibiotic susceptibility^21^. Challenges arise due to the diversity of drug resistance mechanisms, multidrug resistance, and incomplete genome coverage, leading to insufficient sequence reads for detection of ARGs^22^. However, there are some studies that show the potential of SMg for the detection of ARGs^23–25^ by comparing the genotype against the phenotype, or generating AMR profiles from SMg assemblies and comparing them with whole genome sequences (WGS) from isolates. The use of SMg on samples from bone and joint infections has been used where they could predict antibiotic susceptibility in 94.1% (monomicrobial) and 76.5% (polymicrobial) of the cases^15^. However, in these studies, the main obstacle has been a high background of genetic material mainly derived from the host, which generates very few bacterial reads. Similarly, when using SMg data for subtyping bacteria, one of the main challenges is missing loci. This problem arises when coverage is too low to guarantee the presence of a read containing a given sequence in the targeted genome^26^.

We previously showed that SMg on BCBs with PT resulted in acceptable high number of bacterial reads, genome coverage and genome sequencing depth^18^. Here, we wanted to assess the potential of SMg for the identification and typing of the most common cause of PJI, *S. aureus,* and the prediction of virulence and AMR directly from clinical samples.

## Results

### Sequencing data

SMg sequencing from the 19 samples resulted in a mean number of 3,990,292 reads per clinical sample (range 2,608,766–8,086,037). Sequencing of DNA from the sample spiked with *S. aureus* and the negative control produced 5,942,038 and 11,192,852 reads, respectively (Supplementary Table S1). Samples contained a lower proportion of reads classified either as human or horse or PhiX, while 98% of the reads did not map to any of the reference sequences used for the alignment (Supplementary Table S2 and Fig. S1).

**Figure 1:**
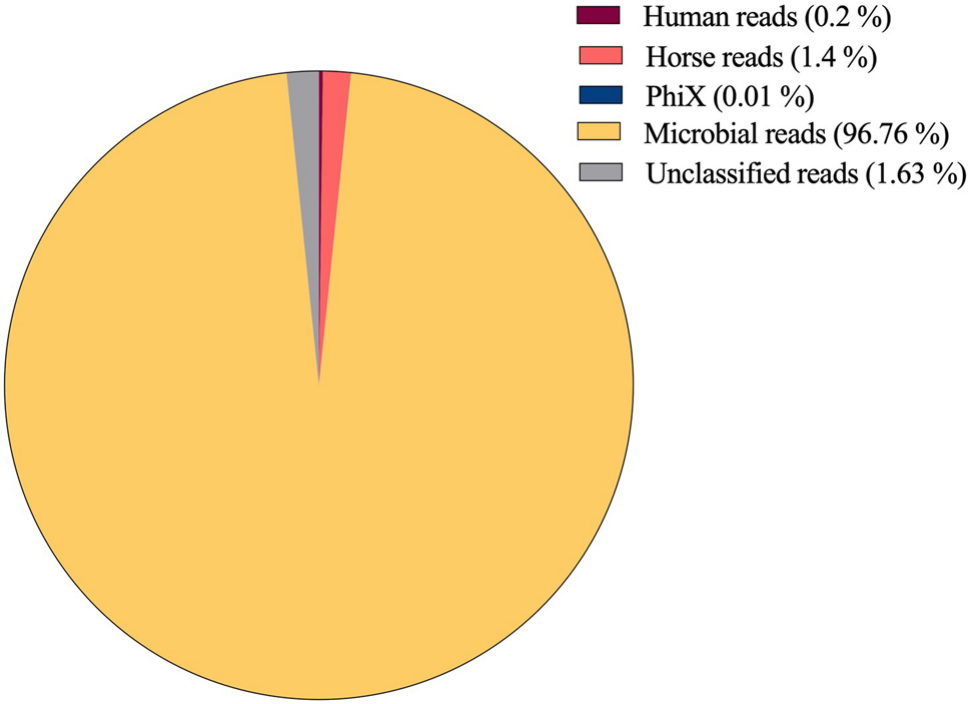
Proportion of reads taxonomically classified as human, horse, PhiX, microbial and unclassified.

After data preprocessing, a mean number of 3,700,731 reads remained for further taxonomical classification while from the negative control only 187,094 reads (1.67%) remained for taxonomical analyses (Supplementary Table S3). Kraken taxonomically classified a mean proportion of 98.36% reads, with 95.74% bacterial reads (Fig. 1 and Supplementary Table S4). Assembly with metaSPAdes yielded a mean number of 232 contigs (range 134–378), with a mean total size of 3.1 Mb (range 2.6–4.8 Mb) in the clinical samples and 213 contigs for a total length of 2.7 Mb in the sample spiked with *S. aureus* (Supplementary Table S5). The total number of base pairs was higher in polymicrobial samples than in monomicrobial ones (4.8 Mb vs 2.7 Mb, respectively, t-test and P-value < 0.0001). The mean of the “maximum contig size” was 262,574 bp (median 264,931 bp, maximum 425,306 bp) in the clinical samples and 218,856 bp in the spiked sample, and no significant difference was observed between polymicrobial and monomicrobial samples (184,461 bp and 283,495 bp, respectively, t-test and P-value = 0.149). Binning with MaxBin in polymicrobial samples grouped a mean number of 43 contigs in the bin assigned to *S. aureus* (range 39–51) with a mean total of 2.6 Mb (range 2.6–2.7 Mb) and a mean maximum length 297,040 bp (234,035–381,826 bp).

### Identification of *S. aureus* by SMg

The taxonomical classification was performed using Kraken on reads and assembled contigs. When the taxonomical classification from the reads identified multiple highly abundant species (polymicrobial samples), contigs were grouped by species into bins, and then used for taxonomical classification. Relative abundance (determined by Bracken) from the most abundant species in the samples varied depending on the selected approach. The bin classified as *S. aureus* was used for downstream analyses for pathogen characterization.

*Staphylococcus aureus* was identified in all the samples by SMg. *S. aureus* (19/19) and *S. agalactiae* (4/19) was identified from both the reads and contigs. *S. aureus* was the most abundant species identified by SMg, with exception of samples 7 and 9, where *S. agalactiae* was more abundant (86.4% and 85.8%, from the reads, respectively) (Table 1 and Supplementary Fig. S1).

**Table 1:** Bacteria identified in the samples and in the positive control (PC) by MALDI-TOF from blood culture bottles (BCBs) and by Kraken from the shotgun-metagenomics (SMg) with their relative abundance (percentage in parentheses) determined by Bracken on the reads, contigs and bins.

Sample ID	Patient no.	Microorganism(s) identified				
		BCBs (MALDI-TOF)	Shotgun-metagenomics			
			Reads	Contigs	Bins	
					No	Taxonomy
1	1	*S. aureus*	*S. aureus* (99.9%)	*S. aureus* (100%)		
2	2	*S. aureus*	*S. aureus* (99.9%)	*S. aureus* (100%)		
3	3	*S. aureus*	S*. aureus* (99.9%)	*S. aureus* (100%)		
4	4	*S. aureus*	*S. aureus* (99.3%)	*S. aureus* (98.6%)		
5	5	*S. aureus*	*S. aureus* (99.9%)	*S. aureus* (100%)		
6	6	*S. aureus*	*S. aureus* (90.9%) *S. agalactiae* (8.5%)	*S. aureus* (60.6%) *S. agalactiae* (39.4%)	1	*S. aureus* (100%)
					2	*S. agalactiae* (100%)
7	7	*S. aureus* *S. agalactiae*	*S. aureus* (10.7%) *S. agalactiae* (86.4%)	*S. aureus* (62.4%) *S. agalactiae* (37.6%)	1	*S. agalactiae* (97%) *S. aureus* (2%)
					2	*S. aureus* (100%)
8	8	*S. aureus*	*S. aureus* (97.6%) *S. agalactiae* (2.2%)	*S. aureus* (51.5%) *S. agalactiae* (48.2%)	1	*S. aureus* (100%)
					2	*S. agalactiae* (100%)
9	9	*S. aureus*	*S. agalactiae* (85.8%) *S. aureus* (11.4%)	*S. agalactiae* (39.8%) *S. aureus* (60.3%)	1	*S. agalactiae* (94.8%)
					2	*S. aureus* (100%)
10	10	*S. aureus*	*S. aureus* (99.8%)	*S. aureus* (100%)		
11	11	*S. aureus*	*S. aureus* (98.2%)	*S. aureus* (98.3%)		
12	12	*S. aureus*	*S. aureus* (99.8%)	*S. aureus* (100%)		
13	13	*S. aureus*	S*. aureus* (99.4%)	*S. aureus* (100%)		
14	14	*S. aureus*	*S. aureus* (99.6%)	*S. aureus* (100%)		
15	15	*S. aureus*	*S. aureus* (99.4%)	*S. aureus* (100%)		
16	16	*S. aureus*	*S. aureus* (99.9%)	*S. aureus* (99.0%)		
17	17	*S. aureus*	*S. aureus* (89.8%)	*S. aureus* (99.2%)		
18	2	*S. aureus*	*S. aureus* (89.7%)	*S. aureus* (97.6%)		
19	1	*S. aureus*	*S. aureus* (99.9%)	*S. aureus* (84.7%)		
PC	NA	*S. aureus*	*S. aureus* (99.9%)	*S. aureus* (95.1%)		

We identified some reads assigned to *S. aureus* (10.3%) in the negative control. The reads were evaluated by mapping them against a *S. aureus* reference genome (accession number GCF_000144955.1). Results showed that 4% of the reads mapped to the reference genome and 6% of the reference genome was covered. When visualizing the mapping results, reads mapped with genetic areas belonging to coding sequences annotated as RNAs with a very low coverage depth, and they were not distributed all over the genomes. Also, other species were found in the NC but in a very low abundance except for the species *Bacillus cereus* (81.5%) which was the most abundant species found. (For further details on the taxa identified in the negative control, see Sanabria et al.^27^).

### *Staphylococcus aureus* antibiotic resistance determinants by SMg

The presence of antibiotic resistance genes (ARGs) was determined by SMg from the reads and contigs for all the samples, and from the bins classified as *S. aureus* in polymicrobial samples (Sample 6, 7, 8 and 9). The presence of ARGs found in the reads, contigs and bins using the the NCBI bacterial antimicrobial resistance reference gene database as reference, were determined, and compared with the results obtained by the phenotypic antimicrobial susceptibility testing (AST) (Table 2, Supplementary Tables S6 and S7).

**Table 2:** Antibiotic resistance genes (ARGs) detected in this study using different approaches (reads, contigs and bins) with the NCBI bacterial antimicrobial resistance reference gene database, chromosomal mutations detected using ResFinder, and antibiotic resistance phenotype observed by the conventional antibiotic susceptibility testing (AST).

Sample no.	Conventional antibiotic susceptibility test	Chromosomal mutations	ARGs detected from shotgun-metagenomics		
			Reads	Contigs	Bins
1	Penicillin		*blaZ, tet38*	*fosB, blaZ, tet38*	
2	Penicillin		*blaZ, tet38*	*blaZ, tet38*	
3		*grlA*	*tet38*	*tet38*	
4	Penicillin			*fosB, blaZ, tet38*	
5		*grlA*	*tet38*	*tet38*	
6	Penicillin	*grlA*		*tet38*	*tet38*
7	Penicillin		*tetM, tet38*	*fosB, tetM, tet38*	*fosB, tet38*
8			*tet38, tetM*	*tet38*	
9	Penicillin		*tet38, tetM*	*fosB, tetM, tet38*	*fosB, tet38*
10		*grlA*		*tet38*	
11				*fosB, tet38, blaZ*	
12	Penicillin Fusidic acid			*fosB, blaZ, tet38*	
13				*blaZ, tet38*	
14	Penicillin		*blaZ*	*fosB, blaZ, tet38*	
15	Penicillin	*grlA*	*blaZ, tet38*	*blaZ, tet38*	
16	Penicillin Fusidic acid		*blaZ, tet38*	*fosB, blaZ, tet38*	
17				*tet38*	
18	Penicillin Fusidic acid	*grlA*		*blaZ, tet38*	
19	Penicillin Fusidic acid		*blaZ, tet38*	*fosB, blaZ, tet38*	
PC			*tet38*	*fosB, tet38*	

The mean coverage depth when predicting ARGs from the reads was 209x (Supplementary Table S6). We applied a threshold of a minimum 20 × coverage depth, 90% sequence identity and 90% sequence coverage for determining the presence of ARGs. In total, we were able to identify three different resistance genes in *S. aureus* (*tet38*, *blaZ* and *fosB*) in the 20 samples (including the spiked sample) by SMg. A higher number of ARGs were detected in the samples using the contigs approach compared with the reads approach (43 and 20 predictions in total, respectively).

The gene *tet38* encoding the chromosomally encoded efflux pump of *S. aureus* was detected across all samples. The other genes detected, corresponded to the *blaZ* gene (35%), and the fosfomycin resistance gene *fosB* (50%). In the polymicrobial samples, the gene *tetM* was also detected, but it was not identified in the *S. aureus* bins.

Resistant phenotypes were observed only for penicillin in 12/19 samples (63.5%), and for fusidic acid in 2/19 (10.5%) samples (Table 2). The penicillin resistant phenotype could be explained by the presence of the *blaZ* gene in most of the samples. However, in samples 6, 7 and 9, the penicillin resistance phenotype could not be explained by the genotypic profile generated from the reads, contigs or bin. The fusidic acid resistance phenotype could not be explained by the results obtained genotypically. Chromosomal point mutations in the genes reported to induce resistance to fusidic acid (genes *fusA* and *fusE)* were not identified. Contrariwise, mutations in the *grlA* gene were observed in six samples (31.6%). Although, no ciprofloxacin resistance phenotype was observed in any of them (Table 2).

### Virulence factors

Overall, a total of 73 genes coding for virulence factors (VFs) were found by SMg in *S. aureus* (Supplementary Table S8) and a mean of 55 virulence genes (range, 50–62) were detected per sample using the VFDB and thresholds of 90% identity and 90% sequence coverage. Toxins, adhesins and immune evasion molecules were among the genes predicted. Genes encoding 40 virulence factors were present in all the samples, e.g., the *cap8* capsule genes (cap8A-G and cap8L-P), iron sequestration operon *isdA*-*isdG,* and exotoxins *hla, hld*, *hlgA*-*C,* among others. Additionally, in five samples the toxic shock syndrome toxin 1 (*tsst-1*) gene was detected.

**Figure 2:**
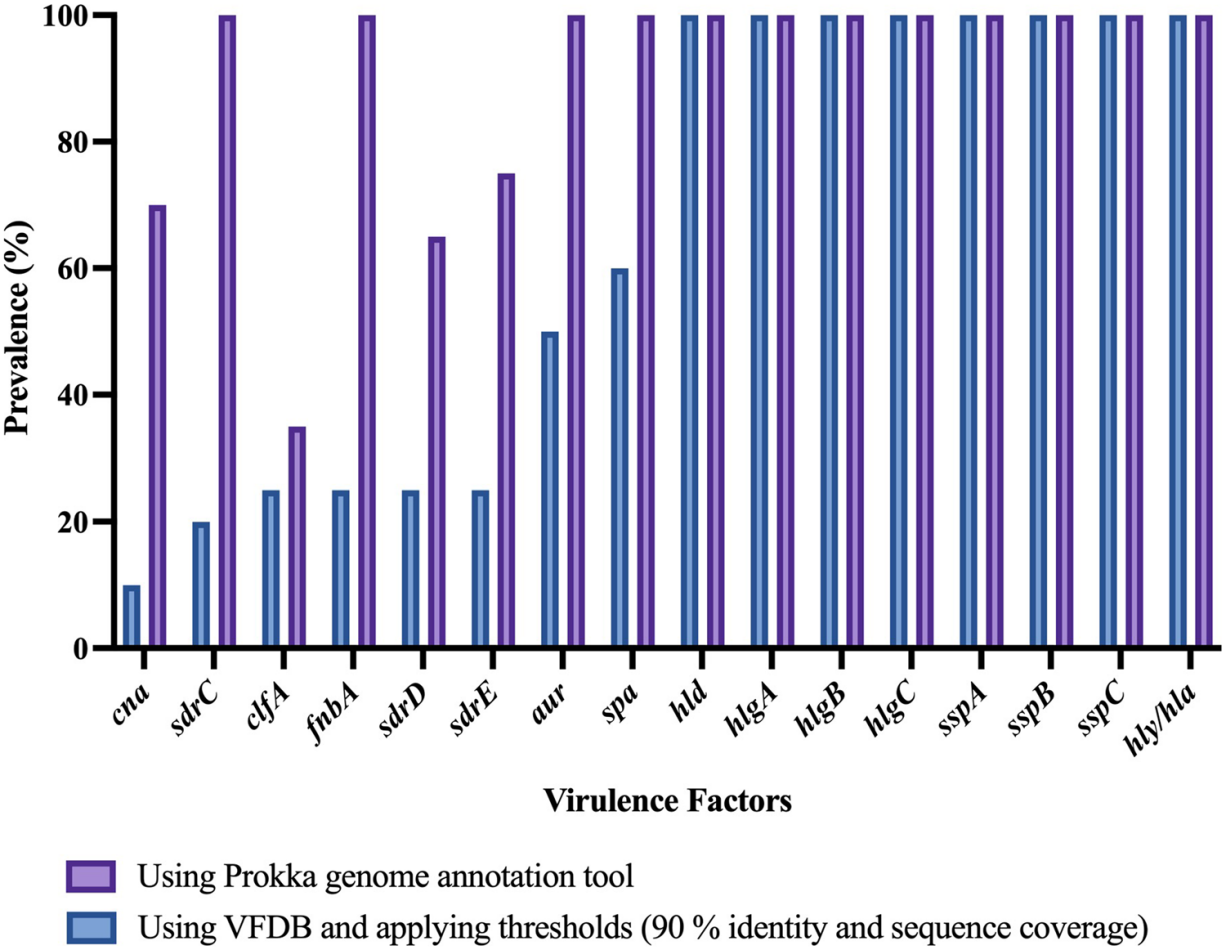
Virulence genes predicted by SMg from *S. aureus* in PT samples in this study*.*

Virulence genes recognized as belonging to *S. agalactiae* were removed from the analysis of polymicrobial samples. Several virulence genes known or proposed to play a role for the pathogenicity of *S. aureus* in PJI were identified from the metagenomes (Fig. 2 and Table 3). Prokka annotation tool was used to confirm the virulence genes presence or absence in the metagenomes. Figure 2 and Table 3 represent the Prokka results on the selected virulence genes found associated with PJI together with the results obtained when using the VFDB applying the thresholds (90% identity and 90% sequence coverage).

**Table 3:** Prevalence of virulence genes known or proposed to play a role in *S. aureus* pathogenicity in PJI predicted from SMg in this study using 90% identity and 90% sequence coverage.

Virulence gene	Product	Approach	
		Prokka annotation	VFDB annotation
*aur*	Zinc metalloproteinase aureolysin	100	50
*clfA*	Clumping factor A fibrinogen-binding protein	35	25
*cna*	Collagen adhesin precursor	70	10
*fnbA*	Fibronectin-binding protein A	100	25
*hld*	Delta-hemolysin	100	100
*hlgA*	Gamma-hemolysin chain II precursor	100	100
*hlgB*	Beta-hemolysin	100	100
*hlgC*	Gamma-hemolysin component C	100	100
*sdrD*	Ser-Asp rich fibrinogen-binding bone sialoprotein-binding protein	65	25
*sdrE*	Ser-Asp rich fibrinogen-binding bone sialoprotein-binding protein	75	25
*spa*	Immunoglobulin G binding protein A precursor	100	60
*sspA*	Serine protease; V8 protease; glutamyl endopeptidase	100	100
*sspB*	Staphopain cysteine proteinase SspB	100	100
*sspC*	Staphostatin B	100	100
*hly/hla*	Alpha-hemolysin precursor	100	100

### MLST and cgMLST analysis

**Figure 3:**
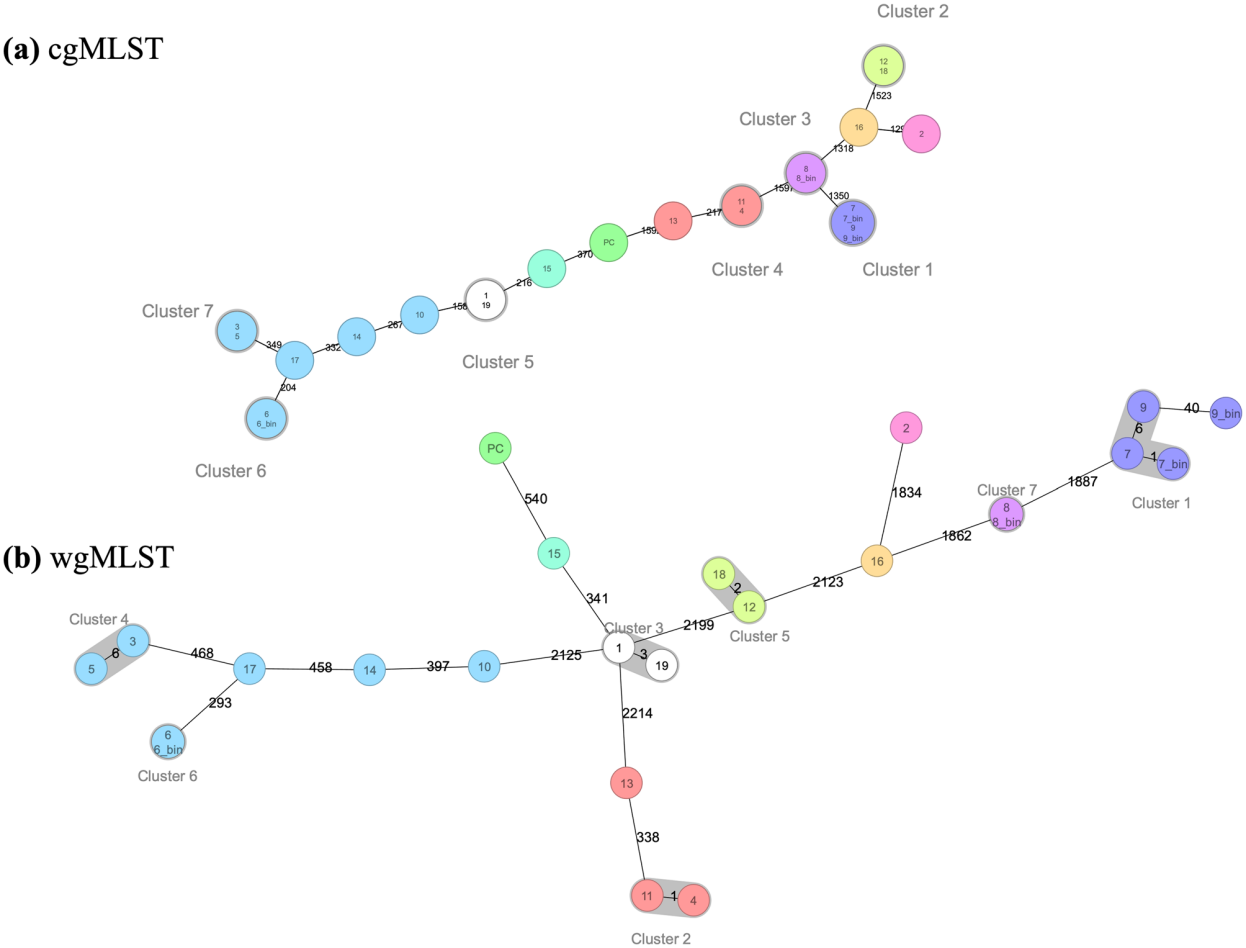
Minimun-spanning tree based on cgMLST (**a**) and wgMLST (**b**) allelic profiles of *S. aureus* genomes obtained from SMg. Color nodes according to sequence type. The number in the connecting lines illustrates the number of targeted genes with differing alleles.

The *S. aureus* Multilocus sequence types (ST) were identified for all the samples at both core genome and whole genome (core genome + accessory genome genes) level (Fig. 3 and Supplementary Table S9). Typing from SMg data showed that *S. aureus* in our samples is of different lineages. *S. aureus* in the samples represent six different clonal complexes (CCs), and they belonged to different STs (ST45 (30%), ST121 (15%), ST30 (15%), ST22 (10%), ST5 (10%), ST15 (5%), ST243 (5%), ST7 (5%) and ST97 (5%)). With respect to polymicrobial samples 7 and 9, typing analyses from both the contigs and the bins classified *S. aureus* as belonging to the same sequence type (ST5). CCs could not be identified for six samples (23%). Samples belonging to the same patient (sample 1 and 18; sample 2 and 19) did not cluster together and did not belong to the same ST (Fig. 3 and Supplementary Table S9). The *S. aureus* isolates analyzed here represent eight phylogenetically diverse STs. cgMLST subdivided the samples into 13 different complex types (CT, 21,308–21,321) (Supplementary Table S9).

## Discussion

Here, we assessed the performance of SMg for the prediction of ARGs, virulence gene determinants and typing of *S. aureus* from clinical PT samples on BCBs. We investigated if there was a difference in outcome from analyzing sequencing data from reads and contigs, and our data analysis followed different analytical approaches in order to identify the procedures that may give the most relevant and accurate information from our SMg data.

It is established that it is possible to analyze sequence data without assembly, but most analyses can be improved by constructing longer contiguous sequences (contigs) through assembly processes^28^. According to our results, the selection of approach to some degree depends on what type of information you require from the data. For instance, if the aim is pathogen identification, taxonomy from the reads is sufficient while if AMR is the focus, the contigs approach will provide a more comprehensive resistome resolution.

For identification of potential pathogen(s) causing PJI, we found that the relative abundance of the taxa was influenced by the approach used to analyze the data. This was evidenced by the differences in abundance obtained, especially in the polymicrobial samples. We observed that the proportion of contigs classified into a certain taxonomical level is also influenced by the genome size. Determining the taxa present and the relative level or number of cells of one taxon vs another (e.g., polymicrobial samples) in a clinical sample is important for identifying the pathogen(s) causing the infection. Our results suggest that analyzing reads provides a more trustworthy representation of the species in a clinical sample than analyzing contigs. Moreover, it is considered that the reads will describe more accurately the proper distribution of species in the sample and provide a more accurate and specific assignment of taxa than using the contigs^29^. This statement applies especially to quantitative community profiling and identification of organisms with close relatives in the database as in the case of clinical microbiology, where the focus is mainly the presence or absence of infectious pathogens, which are well studied and have many completed genomes in the reference databases^30^. In addition, it could also be errors in joining contigs from two closely related species. Specificity is lost when working with contigs as the quality of the assembly will depend strongly on the length and quality of the reads, sometimes misrepresenting the original sample^29^.

Interpretation of SMg results can be challenging, especially when distinguishing microorganisms actually present in the sample from contaminants^31^. In this study, *S. agalactiae* was detected in four samples by SMg, but it was only detected in one of these samples by the laboratory method (BCBs). *S. agalactiae* has been reported as one of the most common pathogens found together with *S. aureus* in polymicrobial PJIs^32^. This could suggest an increased sensitivity of SMg to detect bacteria, or alternatively a contamination problem in the laboratory workflow. These findings highlight the importance of the use of parallel negative controls (no-template control) and DNA spiked positive controls to exclude or identify sources of contamination and minimize false-positive results^33^. Bioinformatic tools have been developed to streamline identification of contaminants and/or cross-contamination, like e.g. Recentrifuge^34^ and Decontam^35^.

*S. aureus* has been studied extensively with a special focus on resistance and virulence. In this study, only resistance to penicillin (63.5%) and fusidic acid (10.5%) were observed by AST. No MRSA isolates were detected, neither by phenotypical nor genotypical testing. This result is not surprising, since Norway has a very low prevalence of MRSA^36^.

Although SMg is a promising approach, the in silico translation from genotype into phenotype relies on the knowledge about the genomic resistance determinants^37^. Our SMg analysis allowed the prediction of the genotypical resistance profile from *S. aureus* present in the samples. The ARG *tet38* was detected in all samples. However, tetracycline resistant phenotypes were not observed, and the presence of *tet38* is not enough to produce a resistant phenotype. The *tet38* determinant is an inherent, chromosomally encoded efflux pump in *S. aureus* and resistance to tetracyclines is often associated with plasmid-mediated genes encoding active efflux pumps or proteins that protect ribosomes from drug action^38^.

In three of the metagenomic samples with phenotypic penicillin resistance, the *blaZ* gene was absent (25%). Similarly, fusidic acid resistance phenotype was observed in two samples (sample 12 and 16). The presence of the *fusB* gene or mutations in *fusA* (encoding EF-G) or *fusE* (encoding ribosome protein L6) genes known to cause fusidic acid resistance were not detected in the metagenomes. Therefore, the phenotype could not be explained by the results obtained genotypically. Overall, interpretation of cases where phenotyping reports resistance but WGS methods predict susceptibility is considered difficult^39^.

Sometimes, detection of ARGs may be challenging due to the variable location of the resistance genes, since some of them can be on a plasmid or integrated into the chromosome, as is the case of the *blaZ* gene and the *fusB* gene which has been found on *S. aureus* plasmid or on phage-related resistance islands integrated into the chromosome^40^. Bacterial isolates with plasmid-encoded copies may have very high (if multiple copies are carried) or very low (because of poor mapping to the reference) coverage in that region^41,42^. As a result, these regions may be rejected as low coverage when predicted from the reads, or as poor quality by the assembly tools when predicted from the contigs and bins since they fall outside the coverage level of the rest of the genome. This problem may be overcome in the future with long-read sequencing methods such as nanopore sequencing or alternative methods for de novo assembly.

Another possibility is the presence of small colony variants (SCV) being present phenotypically but overgrown in WGS culture and thus not represented in the sequence^39^. This phenomenon has been observed for resistance associated with fusidic acid^43^.

We observed that three of the five samples with phenotype-genotype disagreement for penicillin resistance were found to be polymicrobial by SMg (samples 6, 7 and 9). In samples 7 and 9, *S. aureus* was not the most abundant species present in the sample (< 12%) which may affect the prediction of ARGs by a lower sequence coverage.

Prediction of ARGs was done at the reads, contigs and bin level. The total number of different genes detected (3 ARGs) was influenced by the parameters selected to report a gene as present, as these parameters constitute a trade-off between specificity and depth^44^. We used strict parameters, and only ARGs that had ≥ 90% similarity and ≥ 90% sequence coverage to that of the reference were reported from the reads, contigs and bins. In addition, at the read level, only ARGs with at least 20 × coverage depth were considered as present. The selection of ARGs using stringent cutoffs (≥ 90% per read or contig) can increase the probability of targeting genes that are actually functional^44^. However, thresholds should be adapted for certain genes (e.g., *blaZ*, which can be chromosomally integrated or carried on plasmids)^39^.We consider that the high coverage depth (> 200 ×) is an advantage in our SMg approach.

Most of the tools developed for identifying ARGs from metagenomic reads can detect acquired ARGs, but are not able to identify point mutations associated with AMR^45^. The focus here was on acquired resistance since we have used the tools Groot or ABRicate with the NCBI bacterial antimicrobial resistance reference gene database. However, ResFinder was used to detect chromosomal point mutations associated with antimicrobial resistance from the contigs. In our study, more genes were detected at the contig level than at the read level. The detection of more ARGs from the contig-based approach may be explained by the fact that it is easier to reach a high sequence coverage of the gene (90%) from contigs (which are longer) than by reads that are shorter in length. We consider that prediction of ARGs at the contig level is the best approach when looking for ARGs. However, it is important to highlight that both approaches are valid for certain purposes, and both have their limitations. Read-based prediction of ARGs provides an advantage when dealing with metagenomic samples, as ARGs in less abundant organisms can be predicted despite low coverage, which may be missed by assembly-based methods owing to incomplete or poor assemblies^46^. Detecting ARGs from reads is more prone to false positive results because of sequencing errors present in single reads or from DNA contamination from other bacteria. A previous study comparing ARG detection from reads and contigs suggested using both approaches when the sequence coverage is set to a high percentage, since it is possible that ARGs may be separated into different contigs when the number of reads is too low during the assembly process^23^. The use of long-read sequencing can overcome this problem as shown in other studies^17,47–49^. However, there are some limitations for achieving enough bacterial reads to ensure high accuracy when predicting ARGs.

Strategies for predicting AMR phenotypes in polymicrobial samples present an interesting challenge^50^. We tested the binning approach for the prediction of ARGs in *S. aureus* and we found that it gives similar results as prediction of ARGs in monomicrobial samples for most of the genes with exception of the *blaZ* gene. This approach allowed us to separate the contigs belonging to *S. agalactiae* from the contigs belonging to *S. aureus* and predict the *S. aureus* resistance profile, even though there were not many contigs (mean, 44, 3 contigs; range 39–51). This means that AMR genotype predictions could be made from contigs that are binned in a metagenomic assembly, even when they belong to a species that is not in a high abundance. In this study, the binning strategy was no further evaluated. Conclusions from bins should be made with precaution since the binning process can lead to incorrect assumptions due to misbinning (the wrong assignment of a genome fragment from one organism to another), namely if the abundance of the species is very similar, which may lead to neglection of specific determinants^51^.

Apart from antibiotic resistance, virulence of a pathogen is also an important factor clinicians may be concerned about when considering appropriate treatment. Horizontally acquired virulence genes can directly contribute to an infection outbreak, and thus, early identification of virulence factors is important^49,52^. In our study*,* we identified some VFs that are known or proposed to play a role in *S. aureus* PJI, e.g. genes involved in colonization and attachment of host tissue or implanted biomaterials such as the adhesins *clfA* and *fnbA* that encode the fibrinogen and fibronectin-binding proteins, respectively^53^. We have observed that the database used, and the thresholds established to consider the presence of a VF play an important role in the results. The need for a continuously updated curated database is a key challenge for SMg sequencing methods. Thresholds should be adapted for certain genes to improve the prediction and for quality control^39^. Specific VFs may also require the use of different approaches to confirm the results, e.g. to detect genes that are highly polymorphic as the *spa* gene.

MLST was used for strain level typing including a contig-based approach, which means that we had sufficient depth for assembly from the metagenome. wgMLST demonstrated that *S. aureus* in the samples consisted of several lineages. Our MLST results were in accordance with results from the population-based Tromsø Staph and Skin Study, showing that CC30, CC45 and CC15 were the most common CCs in MSSA^54^. Additionally, the most common *S. aureus* lineages in PJI reported in a recent study, were CC30, CC45, CC5, CC15 and CC22. *S. aureus* in our study belonged to the same CCs^32,54^.

Our study has several limitations. First, the sample size was small (n = 19), and we only analyzed a limited number of polymicrobial samples. Second, *S. aureus* isolates were not whole genome sequenced for comparison and confirmation of ARG and VF profiles. Third, no clinical data about the patients were obtained making it difficult to classify the samples as true PJI. Fourth, we have used short-read sequencing which makes detection of ARGs on mobile genetic elements difficult. Long-read sequencing may overcome this limitation. Fifth, we only predicted acquired resistance, which made it difficult explaining the disagreement found between the phenotype and the genotype for penicillin resistance. Errors in sensitivity and specificity of ARG prediction can have different consequences for PJI treatment. False negatives (phenotypically resistant and SMg-susceptible) can lead to inadequate treatment of a resistant infection, increasing morbidity and mortality, whereas false positives (phenotypically susceptible and SMg-resistant) may lead to inappropriate antibiotic use and increase the risk of resistance development^55^. Sixth, we did not include blank negative controls to help identifying genuine sources of contamination coming from the environment or from the reagents. We did not calculate the turnaround time since it was not the focus of the study. However, other studies have estimated the time for SMg using the Illumina technology estimating the total time to 29 h (24–94) from sample extraction to identification and AMR profile^56^. Our protocol would probably also fall into similar turnaround time from BCB positivity to pathogen ID.

Our approach is primarily useful for those using SMg on specimens related to PJI cultured on BCBs, such as synovial fluid, sonication fluid and periprosthetic tissue. It can also be useful for further validation and standardization for the general use of BCBs inoculated with clinical sample. Today, the high costs of our SMg method cannot justify for application on a routine basis^56^. However, in this study, we obtained a high enough sequencing depth, making it possible to multiplex samples and thereby reducing costs considerably.

We do not believe that SMg can replace conventional culturing, but it can be a potential diagnostic tool to support conventional culture in cases when PJI diagnosis is challenging, e.g. fastidious/slow-growing microorganisms, polymicrobial infections, discrepancies between conventional methods, when culture-negative but with clinical sign of infection^57^, and in complicated infections with antibiotic resistant bacteria and long-term antibiotic treatment^58^. In practice, there are no real problems in identifying microorganisms from BCBs. The added value of SMg over classical blood culturing, is mainly that SMg, in addition to pathogen identification, allows extra information in one single procedure, e.g. detecting coinfections, predicting AMR, detecting virulence factors, and bacterial typing. It is still a long way until SMg can be used in the clinical microbiology laboratory. Our SMg approach, including enrichment of microbes using BCBs and human DNA depletion method, has been successful, enabling AMR-prediction, virulence gene detection and bacterial typing.

In conclusion, this study showed that SMg from BCBs inoculated with PT, allowed the identification of potential PJI pathogens and strain-level typing of *S. aureus*. We obtained *S. aureus* ARGs and virulence gene profiles from both monomicrobial and polymicrobial samples. However, the use of this approach for the detection of AMR to help guide clinical treatment needs to be further elucidated, due to the disagreement between the AMR phenotype and genotype. We conclude that the approach chosen for analyzing SMg data (reads, contigs or metagenomic assembled genomes) will have a key impact on the results. Precise AMR prediction is required for mainstream adoption of SMg into the clinical microbiology laboratory. Thus, several improvements are needed for AMR prediction using SMg, including a better understanding of the mechanisms underlying AMR and the procedures (including workflows, tools and databases) that may give the most relevant and accurate information when analyzing SMg data.

## Methods

### Ethics statement

This study was performed in accordance with the ethical guidelines established by UiT—The Arctic University of Norway. The project has been evaluated by the Regional Committee for Medical and Health Research Ethics, REC North, Norway (document no. 2016/1247/REK nord), concluding that ethical approval was not required. According to the Norwegian guidelines, informed consent of the patients is not needed and there were not ethical issues to consider due to the use of anonymous clinical samples and the development of methodological procedures.

### Sample collection

Periprosthetic tissue samples (from hip, knee, elbow, ankle, and shoulder) routinely submitted to the Department of Microbiology and Infection Control at the University Hospital of North Norway (UNN), Tromsø, Norway, were included in this study. Nineteen positive BCBs inoculated with PT from 17 individual patients with suspicion of PJI were used in this study. Clinical samples were selected on the basis of being positive for *S. aureus* by the BCB method^18^*,* either monomicrobial (n = 18) or polymicrobial (n = 1). Samples were collected continuously over a 28-month period (August 2017–December 2019). Samples were anonymized and de-identified. All samples were taken from aerobic bottles (Bact/Alert® FA plus bottles, bioMérieux, Marcy l’Etoile, France).

Five of the 19 clinical samples in this study, in addition to one positive control (spiked sample, BCB inoculated with tissue spiked with *S. aureus* ATCC 2592) and one negative control (DNA not subjected to human DNA depletion, extracted from a BCB enriched with horse blood and inoculated with sterilized tissue from a donor with no suspicion of infection) were obtained from a sample collection in a previous study^18,27^. No blank negative control was included. For further details on the BCB sample preparation method and on the controls, see Sanabria et al.^27^. Bacterial identification was performed using matrix-assisted laser desorption ionization-time of flight mass spectrometry (MALDI-TOF® MS Bruker Daltonics—microflex™). An overview of all the samples included in this study, is shown in Table 1.

### Antibiotic susceptibility testing

Antibiotic susceptibility testing (AST) of *S. aureus* isolates was performed by disc diffusion test according to EUCAST guidelines and the breakpoint table v.10.0 (2020)^59^. The antibiotics tested were penicillin (10 µg), trimethoprim-sulfamethoxazole (25 µg), cefoxitin (30 µg), fusidic acid (10 µg), clindamycin (2 µg), erythromycin (15 µg), linezolid (10 µg), tetracycline (30 µg), norfloxacin (10 µg), ciprofloxacin (5 µg), gentamicin (10 µg) and rifampicin (5 µg) (Oxoid, Basingstoke, UK).

### DNA preparation

DNA was extracted and processed as previously described^18^. In short, all samples were pre-treated using MolYsis™ Basic5 kit (Molzym, Bremen, Germany) to deplete human and horse DNA before DNA extraction using the QIAamp BiOstic Bacteremia DNA Kit (Qiagen, Hilden, Germany). Total DNA concentration was measured using a Qubit dsDNA HS Assay Kit (Life Technologies, Carlsbad, CA, USA) and DNA quality was determined by Nanodrop.

### Metagenomic sequencing

Sequencing libraries were prepared as previously described^18^, using the ThruPLEX® DNA-seq Kit (Rubicon Genomics, USA) following the manufacturer’s instructions. Approximately 100 ng of DNA was used as input for library preparation from the clinical and spiked samples. The sequencing process was performed at the Norwegian Sequencing Centre, Oslo, using a MiSeq sequencer (Illumina Inc., San Diego, CA, USA) with v2 chemistry and 500 cycles for 250 bp paired-end sequencing. Samples were multiplexed with four samples per lane.

### Bioinformatic data analysis

**Figure 4:**
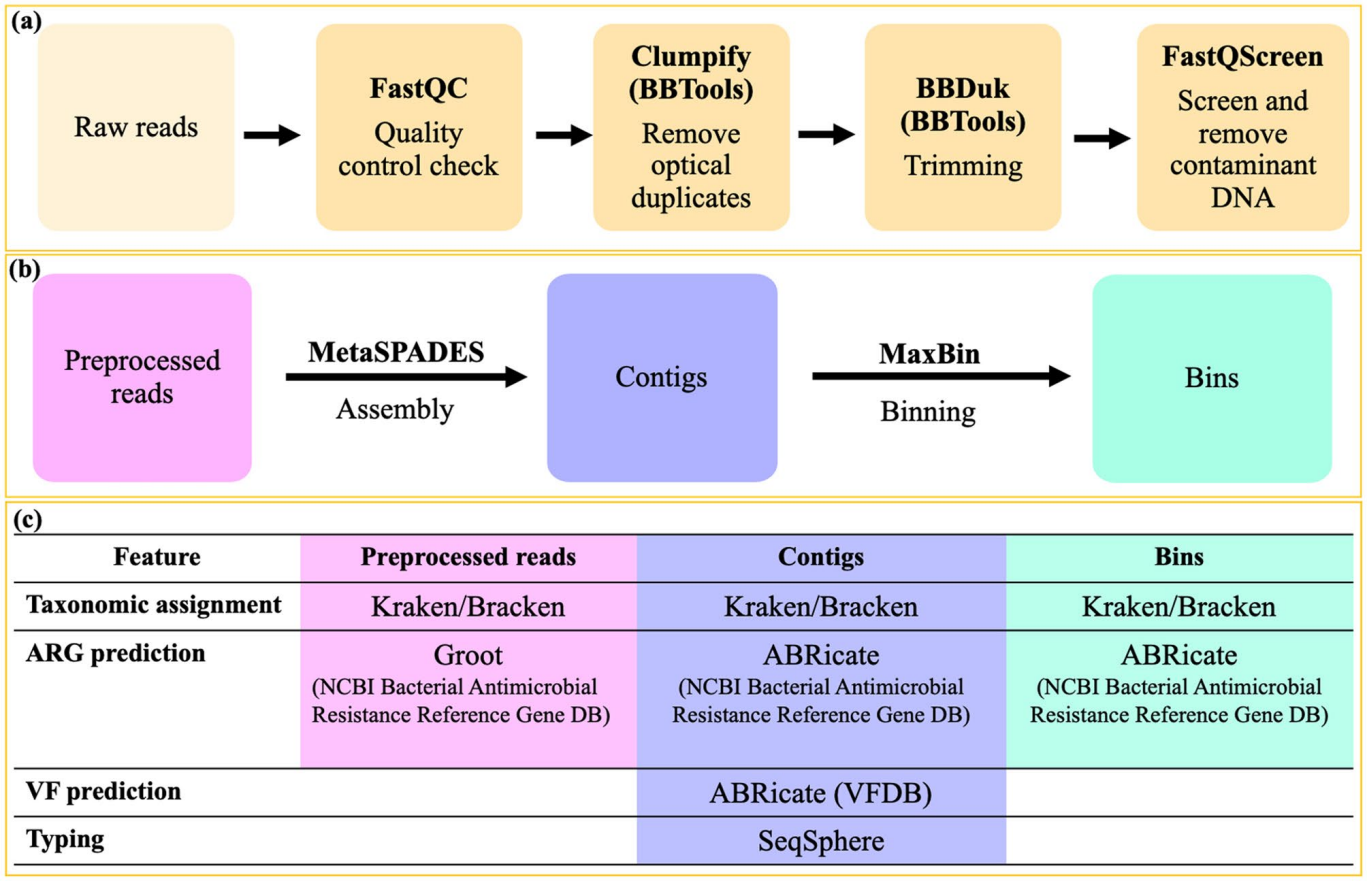
Workflow summarizing the bioinformatic analyses in this study, including (**a**) data preprocessing, (**b**) data analyses approaches and (**c**) data analyses and interpretation. *ARG* antimicrobial resistance gene, *VF* virulence factor, *AMR* antimicrobial resistance.

Our SMg bioinformatics pipeline was created and optimized, based on publicly available tools and pipelines and tools from other SMg studies^9,11–13,15,23,60,61^. The bioinformatic analysis followed in this study is summarized in Fig. 4. The quality of the raw reads in fastq format was assessed using FastQC software v0.11.8 (http://www.bioinformatics.babraham.ac.uk/projects/fastqc/). Optical duplicates were removed using the program Clumpify v38.36 from BBTools suite (https://jgi.doe.gov/data-and-tools/bbtools/) with default parameters. Adapter sequences were trimmed off and the poor-quality reads were removed using BBDuk of BBTools suite. The minimal read length and Phred score were set to 50 nucleotides and 20, respectively. In order to filter out known sources of contaminant host DNA, the reads mapped against the reference genomes of human GRCh38.p13 (GCF_000001405.39), horse (GCF_002863925.1) and the PhiX phage (Escherichia virus phiX174, GCF_000819615.1) aligning with Bowtie2 in the tool FastQ Screen v0.13.0^62^. Unmapped reads were used in subsequent analyses.

Data analyses in this study followed different approaches: using the preprocessed reads directly and using assembled contigs or bins for monomicrobial and polymicrobial samples, respectively. Reads were assembled into contigs using metaSPAdes^63^ from SPAdes v.3.14.0^64^ and the quality of the assemblies was assessed using QUAST v5.0.2^65^. Resultant contigs were annotated using Prokka v.1.13 (http://github.com/tseemann/prokka). Contigs can be grouped by species into discrete units, referred to as bins, which were predicted using the tool MaxBin v.2.2.7^66^, for recovering the *S. aureus* genome from the metagenomic datasets in polymicrobial samples.

Species identification on reads, contigs and bins was performed using Kraken v1.1.1^67^ (as illustrated in Fig. 4) and the 8 GB DustMasked MiniKraken database (as of Oct. 18, 2017) with default parameters. Species relative abundance was determined using Bracken^68^. The detection of antimicrobial resistance genes (ARGs) from the reads was determined using the tool Groot v.1.0.2^69^ (https://github.com/will-rowe/groot). ARGs and virulence genes from the assembled contigs and bins were detected using ABRicate v0.8 (https://github.com/tseemann/abricate). For the detection of ARGs with Groot and ABRicate the NCBI bacterial antimicrobial resistance reference gene database (BioProject accession number PRJNA313047, as of April 24^th^, 2020) was used. Moreover, chromosomal point mutations associated with antimicrobial resistance were identified with ResFinder v.4.1 (Point Finder database as of Feb. 1, 2021). For detection of virulence genes, the virulence factor database (VFDB) was used^70^. The thresholds used for determining the presence of ARG genes and VFs were set as 90% identity and 90% sequence coverage. Additionally, for ARGs prediction from the reads, a coverage depth of at least 20 × was considered to report an ARG as present. Presence or absence of several VFs in the metagenomes, known or proposed to play a role in *S. aureus* pathogenicity in PJI were confirmed using the results from Prokka v.1.13. We used default parameters for all the bioinformatic tools unless it is stated.

### Typing

The assembled contigs and the bins were imported into SeqSphere + software v.6.0.2 (Ridom GmbH, Münster, Germany) for a gene-by-gene allele calling comparison using the *S. aureus* species-specific scheme within SeqSphere + for a cgMLST scheme for comparison of the 1,816 core loci in *S. aureus*, and an accessory typing scheme (wgMLST) with 706 accessory loci. Loci that flagged as failed (i.e., found but bearing frameshifts, or a differing consensus sequence, or having too-low coverage) were considered absent. Phylogenetic trees were constructed in SeqSphere + using a minimum-spaning tree; missing values were pairwise ignored. The cluster-alert distance was set at a default of 24 allelic differences^32^.

### Statistical analysis

Descriptive statistics for categorical variables were based on percentages and frequencies and was used to determine the prevalence of the ARGs and VFs, while continuous variables were based on means, medians, and interquartile ranges (IQRs) and was used to describe the results obtained from the sequencing data. In addition, t-test was used to evaluate if the differences between the total number of base pairs in polymicrobial and monomicrobial samples were statistically significant. The differences were considered statistically significant with p values < 0.0001. Data were analyzed utilizing GraphPad Prism software, version 8.3.0 (GraphPad Software Inc., CA, US).

## Supplementary Information

Supplementary Legends.

Supplementary Figures.

Supplementary Tables.

## Data Availability

The preprocessed reads generated for this study for each sample can be found in the European Nucleotide Archive (ENA) repository (www.ebi.ac.uk/ena) under the project number PRJEB43858.
