# Supplementary material for: Shotgun-metagenomics based prediction of antibiotic resistance and virulence determinants in Staphylococcus aureus from periprosthetic tissue on blood culture bottles
Source: Sci Rep. 2021 Oct 21;11:20848. doi: 10.1038/s41598-021-00383-7 (PMC8531021; doi:10.1038/s41598-021-00383-7)
Supplement: Supplementary file 1 — Supplementary Legends. [file 41598_2021_383_MOESM1_ESM.docx]

**Supplementary material legends**

- **Supplementary Table S1.** Raw data basic statistics.
- **Supplementary Table S2.** Results from the mapping against the known sources of contamination.
- **Supplementary Table S3.** Results from all the preprocessing steps.
- **Supplementary Table S4.** Kraken classified reads.
- **Supplementary Table S5.** Assembly statistics obtained from the assessment of the assembly using QUAST
- **Supplementary Table S6.** Antimicrobial resistance genes predicted from reads using the NCBI Bacterial Antimicrobial Resistance Reference Gene Database and depth coverage for the resistance genes.
- **Supplementary Table S7.** Antimicrobial resistance genes predicted from contigs using the NCBI Bacterial Antimicrobial Resistance Reference Gene Database.
- **Supplementary Table S8.** Virulence factors list.
- **Supplementary Table S9.** Results from MLST using SMg
- **Supplementary Figure S1.** Microorganisms identified by SMg in this study. (A) Percentage of reads classified by Kraken and (B) Species relative abundance re-estimated by Bracken.
