# Supplementary figures and images for: Shotgun-metagenomics based prediction of antibiotic resistance and virulence determinants in Staphylococcus aureus from periprosthetic tissue on blood culture bottles
Source: Sci Rep. 2021 Oct 21;11:20848. doi: 10.1038/s41598-021-00383-7 (PMC8531021; doi:10.1038/s41598-021-00383-7)

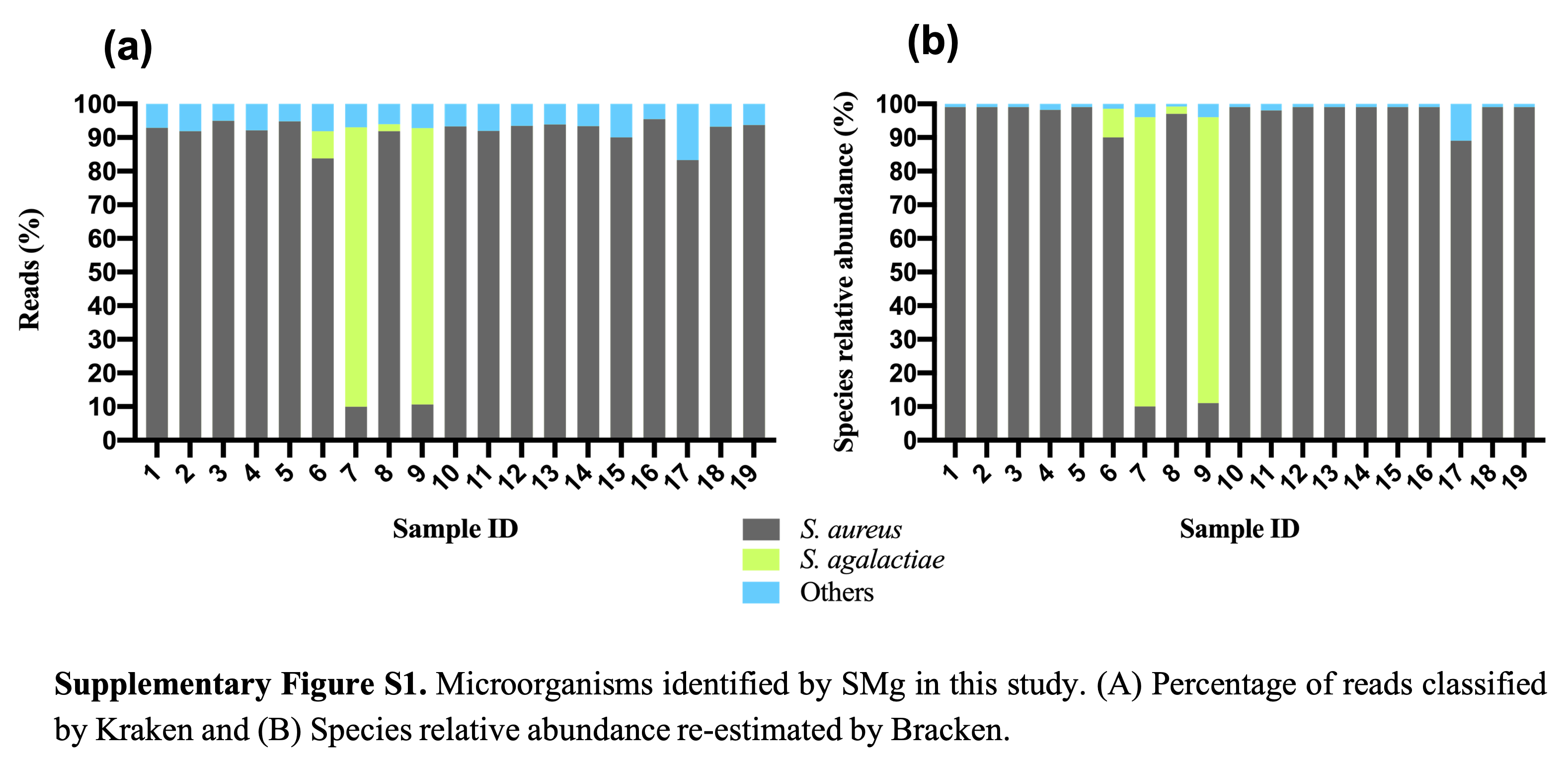

Supplement: Supplementary file 2 — Supplementary Figures. [file 41598_2021_383_MOESM2_ESM.png]
